# Acute retroperitoneal hemorrhage after oocyte retrieval: a case report

**DOI:** 10.1093/jscr/rjab066

**Published:** 2021-03-13

**Authors:** Yangying Xu, Cuifang Hao, Xiaoqiang Liu, Zongzhi Yang, Xianhua Sun

**Affiliations:** Reproductive Medicine Center of Women and Children's Hospital affiliated to Qingdao University, Qingdao 266000, Shangdong, China; Reproductive Medicine Center of Women and Children's Hospital affiliated to Qingdao University, Qingdao 266000, Shangdong, China; Reproductive Medicine Center of Women and Children's Hospital affiliated to Qingdao University, Qingdao 266000, Shangdong, China; Reproductive Medicine Center of Women and Children's Hospital affiliated to Qingdao University, Qingdao 266000, Shangdong, China; Reproductive Medicine Center of Women and Children's Hospital affiliated to Qingdao University, Qingdao 266000, Shangdong, China

## Abstract

A 29-year-old woman with a 5-year history of primary infertility underwent in vitro fertilization-embryo transfer (IVF-ET) treatment. Hemorrhagic shock caused by retroperitoneal hematoma after oocyte retrieval was treated promptly by the evaluation of diagnostic laparoscopy and angiography. The patient was recovered and discharged from the hospital 7 days later without any complications. She was later diagnosed with Von Willebrand disease by a hematologist.

## INTRODUCTION

The first transvaginal ultrasound-guided oocyte retrieval has been performed since 1983 [1], It becomes the most common operation in invitro fertilization-embryo transfer (IVF-ET) treatment and gradually replaces the technique of oocyte retrieval by laparoscopy. It is convenient and safe but there are still rare potential complication, such as infection and bleeding sometimes might even be life threatening. Paolo reviewed 23,827 oocyte retrievals and calculated per retrieval, the overall complication rate was 0.4% [2]. Siristatidis found that the incidence of bleeding after vaginal oocyte retrieval is 0.36%–18.08%, mainly including vaginal bleeding (18.08%), intraperitoneal bleeding (0.36%), retroperitoneal bleeding is extremely rare [3]. Here we report an emergency case of acute retroperitoneal hemorrhage after oocyte retrieval.

## CASE PRESENTATION

A 29-year-old nulligravida woman underwent assisted reproductive technique in our hospital because of 5-year history of infertility. The patient medical history was bilateral obstruction, she had no obstetrics history and had a regular menstruation, normal endocrine profile, no personal and family history. Her blood coagulation profile, blood routine and biochemical tests were normal.

She started to ovulate with gonadotropin (Recombinant Human Follitropin Alfa for Injection) and antagonist (Cetrorelix Aacetate powder for Injection). a scheduled oocyte retrieval via vaginal ultrasound under the intravenous anesthesia after 36 hours of human chorionic gonadotophin administration. Nine oocytes were obtained, everything went well in the procedure. About fifteen minutes later, the cardiogram monitor showed a rapid increase in heart rate to 120–150 per minute and the blood pressure was dropping from 120/70 mmHg to 85/40 mmHg. The patient was still in sedation with anesthesia, she was appeared to be pale and could be waken up complaining abdomen pain and vomiting. Her abdomen was distended and diffusely tender, there was no significant rebound in the lower abdomen, no obvious mobile dullness. Vaginal examination revealed that there is no bleeding in the puncture point, the posterior fornix of the vagina is full and tender. Transvaginal sonography revealed a 3-4 cm free fluid in pelvic, blood gas analysis revealed her hemoglobin concentration was 7.6 g/L. She was immediately given 1 gram of intravenous tranexamic acid, 1 unit Hemocoagulase Atrox for injection and 2 unit of packed red blood cell. Preoperative preparation has been done and the patient agree to laparoscopy procedure.

**Figure 1:**
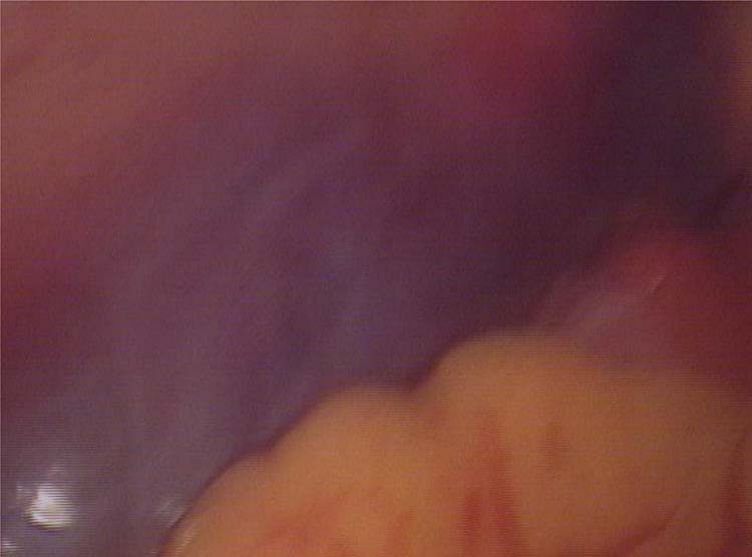
Laparoscopic view of the retroperitoneum hematoma.

Laparoscopy revealed about 200 ml pelvic free fluid, there was no evidence of active bleeder from pelvic vessels, a retroperitoneal hematoma 6-7 cm in diameter up to the left iliac fossae down to 5 cm above the uterine rectal fossae (Fig. 1). The sigmoid colon and its mesenteric membrane are closely adhered to the left lower abdominal wall. The uterus is anterior full in shape and normal in size, the posterior wall of the uterus partly adhered to the rectum, the fallopian tubes are normal in shape.

**Figure 2:**
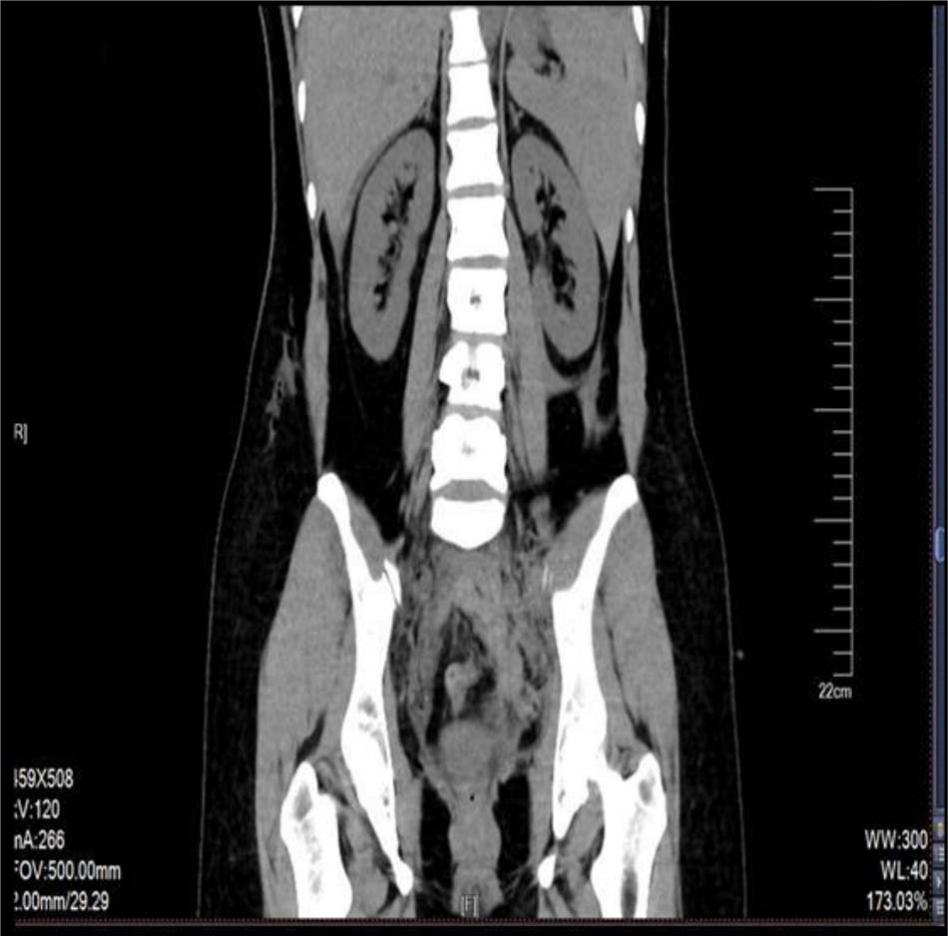
CT scan of the retroperitoneum hematoma.

**Figure 3:**
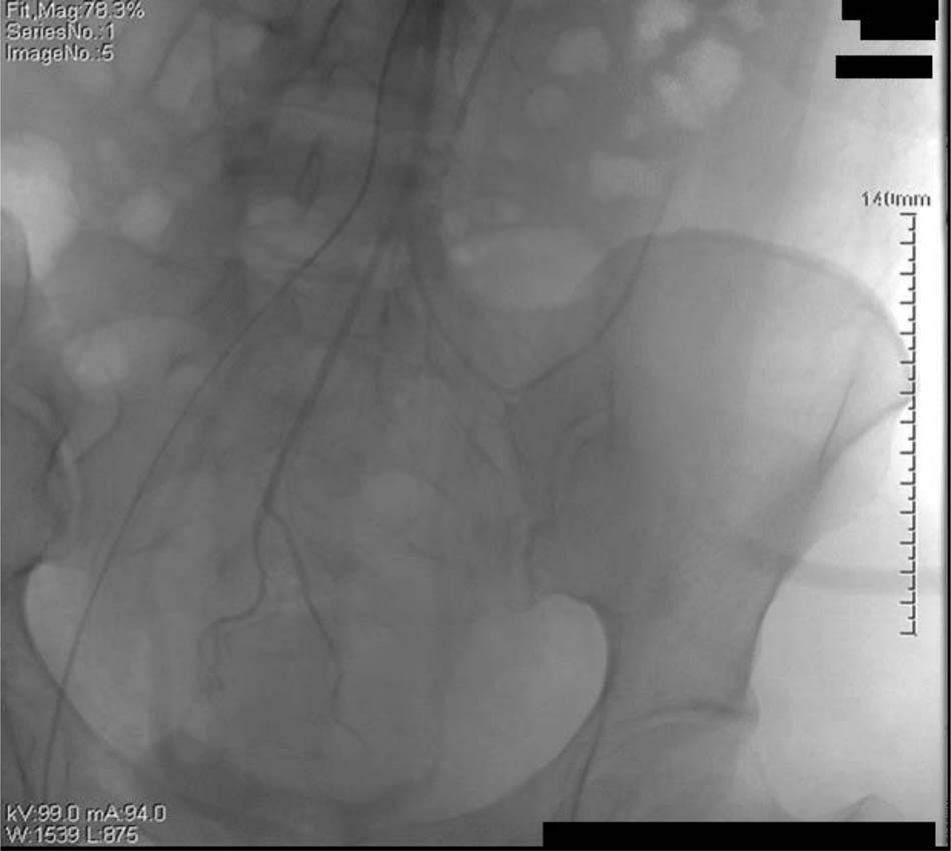
The left iliac artery and inferior mesenteric artery was intact by fluorescence angiography.

The retroperitoneal hematoma appeared stable and no expanding, also no increase in tension. The hemoglobin concentration reexamination showed no significant change. Considering the bleeding has stopped, the trauma surgeon recommended no further incision for the retroperitoneal hematoma after consultation. Digital subtraction angiography (DSA) was done after operation to further confirm no more artery bleeding. DSA revealed the inferior mesenteric artery and the iliac artery were intact (Figs 2 and 3). The patient was placed in the intensive care unit with a angiographic catheter retained for emergency use. They estimated the total blood loss 1500-2000 ml, 200 ml of fresh frozen plasma and 4 units of packed red blood cells were transfused during operation.

She was discharged from hospital after seven days without any complication. The patient recovered well and the retroperitoneal hematoma disappeared within one month. She was referred to see hematologist and was diagnosed with Von Willebrand disease (VWD).

## DISCUSSION

To our knowledge this is the first emergent case of hemorrhagic shock with retroperitoneum hemorrhage after oocyte retrieval, a case had been managed conservatively with retroperitoneal hematoma during laparoscopy. Two previously reported cases of retroperitoneal hemorrhage occurred after oocyte retrieval. Foad Azem reported a 42-year-old woman with retroperitoneal hematoma 7 cm in diameter 6 hours after oocyte retrieval. Laparoscopy revealed the injury of the mid sacral vein. The hematoma was removed and a mental clip was used to control the active bleeding [4]. Mata reported another 36-year-old woman with a vaginal septum resected developed vaginal hemorrhage and retroperitoneal hematoma about 1500-2000 ml blood loss after oocyte retrieval [5]. The retroperitoneal hematoma tracked from the lacerated posterior fornix to the back of the uterus (because of the vaginal laceration extended to the back of the uterus). when the vaginal laceration was repaired, the bleeding was controlled.

Aragona reported that clinical factors related the bleeding complication maybe the factor IX deficiency, ovarian necrotizing vasculitis and anticoagulant treatment, surgical experience is also an important factor [6]. VWD is a disorder that makes it hard for blood to clot because there is not enough of a clotting protein called Von Willebrand factor and blood coagulation factor VIII. The patient we reported has a normal coagulation profile, but an important point in her history supplement has been mentioned that it has taken 20 minutes of pressure to stop the bleeding after every blood drawing. Laparoscopy revealed the left ovary partly adhered to the rectum and multiple endometriosis, the abnormal pelvic anatomy and the junior operator may led to the abnormal puncture path and pelvic retroperitoneal blood vessel lesions.

The management of retroperitoneal hematoma is quite challenging. It is not recommended except the organic injury, the increase tension or with a pulse of the hematoma [7]. Otherwise it is difficult to find the source of bleeding and the chance of infection is greatly increased. The patient we reported with stable and no increase in expand and tension. She was treated conservatively after CT and angiography, she recovered and discharged within 7 days.

In conclusion, although transvaginal ultrasound oocyte retrieval is a general technology in assisted reproductive technology, It is necessary to monitor patients closely after oocyte retrieval for physician. Any changes in vital signs and complaints of abdominal discomfort should be taken seriously, Even there is no significant pelvic free fluid, retroperitoneum bleeding should also be considered especially to those people with abnormal coagulation.

## CONFLICT OF INTEREST STATEMENT

None declared.

## FUNDING

Supported by Qingdao Key Health Discipline Development Fund.
